# Potentially modifiable risk and protective factors affecting mental and emotional wellness in pregnancy

**DOI:** 10.3389/fnhum.2024.1323297

**Published:** 2024-02-20

**Authors:** Fiona Wohrer, Helen Ngo, Jared DiDomenico, Xingya Ma, Melissa H. Roberts, Ludmila N. Bakhireva

**Affiliations:** ^1^School of Medicine, University of New Mexico Health Sciences Center, Albuquerque, NM, United States; ^2^College of Pharmacy Substance Use Research and Education Center, University of New Mexico Health Sciences Center, Albuquerque, NM, United States

**Keywords:** mental health, social support, pregnancy, substance use, alcohol, cannabis

## Abstract

**Introduction:**

Impaired mental and emotional wellness often co-occurs with prenatal substance use, and both affect infant socio-emotional, cognitive, language, motor, and adaptive behavioral outcomes. Guided by the modified biopsychosocial framework, this study examined the role of common substance exposures during pregnancy (i.e., alcohol and cannabis), socio-cultural factors (social support during pregnancy, adverse childhood experiences), and reproductive health factors on maternal mental health (MMH).

**Methods:**

Data were obtained from a prospective cohort study–Ethanol, Neurodevelopment, Infant, and Child Health (ENRICH-2), and included 202 pregnant persons. Alcohol and cannabis exposures were assessed through repeated prospective interviews and a comprehensive battery of drug and ethanol biomarkers. MMH outcomes were evaluated during the third trimester through the Perceived Stress Scale, Edinburgh Depression Scale, Generalized Anxiety Disorders-7, and Post-traumatic Stress Disorder Checklist for Diagnostic and Statistical Manual of Mental Disorders. Univariate and multivariable linear regression models evaluated significant predictors of MMH.

**Results:**

Results of multivariable analysis indicate that both maternal adverse childhood experiences and alcohol exposure, even at low-to-moderate levels, during pregnancy were associated with poorer scores for most MMH measures, while higher level of social support and Spanish as the primary language at home (as a proxy of enculturation) had protective effects (all *p’s* < 0.05).

**Conclusion:**

These findings highlight the importance of assessing substance use, including periconceptional alcohol exposure, and mental health in pregnant persons as closely related risk factors which cannot be addressed in isolation. Our findings also emphasize a strong protective effect of socio-cultural factors on maternal mental and emotional wellbeing—a strong precursor to maternal-infant bonding and infant neurodevelopment.

## Introduction

The World Health Organization (WHO) considers disordered maternal mental health (MMH) to be a major public health challenge globally (World Health Organization, 2016). The prevalence of clinically diagnosed mental health disorders, specifically depression and anxiety, in the perinatal period is estimated to be 11% and 13%, respectively (Falah-Hassani et al., 2017; Smythe et al., 2022). These numbers are likely to substantially underestimate the true prevalence due to underdiagnosis. Furthermore, national data suggests that rates of MMH diagnoses are increasing. A recent analysis from the Centers for Disease Control and Prevention (CDC) indicates a seven-fold increase in diagnosis of depressive disorders at the time of delivery from 2000 to 2015 (Haight et al., 2019). Although the American College of Obstetricians and Gynecologists (ACOG) recommends screening for mental health conditions during routine prenatal care, one in five women report not being asked about depressive symptoms (Bauman et al., 2020; ACOG, 2023). In addition to directly effecting maternal wellbeing, MMH disorders are associated with adverse neonatal outcomes, including preterm birth, low birth weight, poor breastfeeding outcomes, disrupted mother-child bonding, and impaired cognitive and behavioral outcomes in children (Grigoriadis et al., 2013; Davies et al., 2020; Salameh and Hall, 2020; Solé et al., 2020; Simonovich et al., 2021). A meta-analysis, incorporating the data from 191 studies with a combined sample size of 195,751 birthing parent-child dyads, reported a robust and long-lasting association between maternal depression and anxiety during pregnancy and poorer offspring social-emotional, cognitive, language, motor, and adaptive behavioral outcomes (Rogers et al., 2020). The adverse effects were seen across infancy into childhood and adolescence highlighting the importance of the birthing parent’s mental and emotional wellness, as a potentially modifiable factor, for long-term child neurodevelopment.

Substance use in the antenatal period is associated with well-established adverse perinatal outcomes. Alcohol is a known teratogen associated with a plethora of structural and functional defects, collectively known as Fetal Alcohol Spectrum Disorder (Williams and Smith, 2015). Between 2018 and 2020, 13.5% of pregnant women reported current alcohol use and 5.2% reported binge drinking in the past 30 days; both rates increased from previous surveys conducted by the CDC (Gosdin et al., 2022). While prenatal alcohol exposure (PAE) has previously been associated with adverse MMH outcomes, the influence of maternal alcohol use in the periconceptional period as well as the level of alcohol exposure thought to significantly impact MMH are not well documented and thus, represent an important knowledge gap (Zuckerman et al., 1989). Cannabis (marijuana), alcohol, and tobacco are the most frequently used substances among pregnant women (Wendell, 2013). The ACOG and the American Academy of Pediatrics (AAP) recommend abstinence from cannabis use during pregnancy due to scientifically plausible concerns (ACOG, 2017; Ryan et al., 2018). The Substance Abuse and Mental Health Services Administration (SAMHSA) estimates the prevalence of prenatal cannabis use between 3% and 7% nationally (Substance Abuse and Mental Health Services Administration [SAMHSA], 2019). However, other studies estimate cannabis use rates as high as 28% among certain populations of pregnant women (Beatty et al., 2012; Young-Wolff et al., 2019). Cannabis use among pregnant persons increased in recent years, possibly due to stressors imposed by the COVID-19 pandemic and wider legalization of use (Young-Wolff et al., 2021). Legislative changes resulting in increased accessibility of higher potency cannabis products have prompted concerns about potential impacts on maternal and child health (Gnofam et al., 2020; ElSohly et al., 2021).

Prior studies have documented co-occurrence of impaired MMH and substance use (Pentecost et al., 2021). For example, a recent systematic review and meta-analysis demonstrated that the prevalence of postpartum depression (PPD) among women with prenatal substance use is markedly higher than the estimated rate of PPD among the general population (29% vs. 17%) (Pacho et al., 2023). Although antenatal substance use and impaired mental health are frequently co-occurring, their respective repercussions on obstetric and neonatal outcomes are often investigated as independent risk factors. The impacts of maternal social, biological, and reproductive characteristics have been examined in a similarly isolated manner. Many of these risk factors are potentially modifiable, indicating a viable leverage point for intervention. Thus, the scope of consideration must be broadened to capture the complex interplay and interactive potential of risk and protective factors affecting MMH outcomes. Several models have been proposed to categorize an aggregate of factors which affect MMH (Dunkel Schetter, 2011; Franks et al., 2017; Blount et al., 2021). Biopsychosocial theory was proposed to comprehensively characterize the development of psychiatric disorders based on three major dimensions: biological, social and psychological (Tripathi et al., 2019). Interpreting this in the context of the prenatal period requires adjustments for risk and protective factors specifically relevant to the state of pregnancy. Therefore, this analysis proposes a modified biopsychosocial archetype with health domains relevant to maternal experience during pregnancy: *biological, social*, and *reproductive health* (described in detail below). Utilizing this framework, this study aims to comprehensively evaluate the factors influencing MMH in pregnant patients with a specific emphasis on the most prevalent substances used during pregnancy (alcohol as the primary exposure of interest and cannabis as secondary) and the level of maternal social support as a potentially modifiable protective factor (Wendell, 2013; Bedaso et al., 2021).

## Materials and methods

### Conceptual framework

**FIGURE 1:**
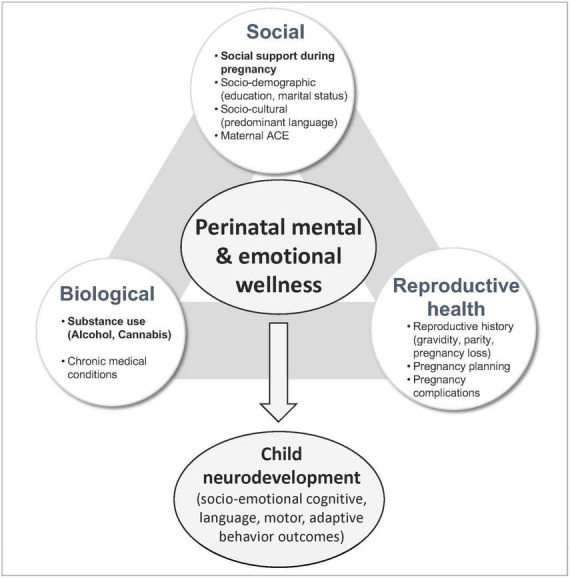
Conceptual framework for risk and protective factors affecting perinatal mental and emotional wellness.

Figure 1 presents a conceptual framework which guided analyses presented in this manuscript. It organizes strategically selected variables into a three-pronged approach for the purposes of defining predictors of MMH. While each domain is distinct, overlap exists between categories due to their cognate nature. The *social domain* incorporates socio-demographic (i.e., education, marital status) and socio-cultural (i.e., predominant language spoken at home as a proxy of enculturation) characteristics, as well as the level of social support during pregnancy, and maternal experiences of adversity during childhood. Social support is defined as the extent to which social relationships fulfill an individual’s needs (e.g., emotional, cultural, affection) and reflect the individual’s current state at the time of evaluation (Sherbourne and Stewart, 1991; Jacobson et al., 2002). Low social support index has been associated with depression, anxiety, and self-harm in pregnant persons (Bedaso et al., 2021). Measures of adverse childhood experience (ACE) reflect maternal socio-environmental landscape during childhood, and thus, are categorized under the social domain (Osofsky et al., 2021). Maternal experiences of household dysfunction and mistreatment during childhood have been associated with higher stress levels during pregnancy and other adverse mental health outcomes (Osofsky et al., 2021; Racine et al., 2021). The *reproductive health domain* encompasses prior experiences related to pregnancy and motherhood which have a distinct impact on maternal psychological wellbeing in the setting of pregnancy (Lagadec et al., 2018). It includes standard reproductive history variables, i.e., parity (number of liveborn children), prior pregnancy loss (miscarriage, spontaneous abortion, ectopic pregnancy, termination), current obstetric complications (bleeding, gestational hypertension/preeclampsia, gestational diabetes, placenta previa) and whether the current pregnancy was planned. These variables have been associated with impaired MMH outcomes in prior studies (Bellieni and Buonocore, 2013; Gariepy et al., 2017; Jairaj et al., 2019; Herbert et al., 2022). The *biological domain* captures the presence of chronic medical conditions (hypertension/heart disease, diabetes, thyroid disorder, asthma/allergies, hepatitis, autoimmune disorder) and maternal substance use. Chronic disease has been associated with mental health disorders in the antepartum and postpartum periods as well as in the general population (Brown et al., 2018; Tsakiridis et al., 2019). Impaired MMH is thought to relate bidirectionally with prenatal substance use and has been independently associated with chronic medical conditions, obstetric complications and disrupted infant neurodevelopment (Rogers et al., 2020; Salameh and Hall, 2020).

### Study design and population

Data for this study was obtained from the Ethanol, Neurodevelopment, Infant, and Child Health (ENRICH-2) prospective cohort study conducted at the University of New Mexico (UNM) in 2018–2023 (Maxwell et al., 2023). The study was approved by the Human Research Review Committee; all participants provided informed consent in writing. Patients primarily in their second trimester of pregnancy were screened and recruited from UNM affiliated prenatal care clinics. The following eligibility criteria were applied: (1) at least 18 years of age; (2) singleton pregnancy; (3) planning to deliver at UNM hospital; (4) planning to reside in New Mexico for at least 6 months after delivery to complete all study visits; and (5) ability to provide written consent in English or Spanish.

The study included four prospective visits. Briefly, Visit 1 was conducted during a routine prenatal visit and consisted of an eligibility screen, a baseline interview, and collection of maternal blood and urine samples. Visit 2 was conducted during the third trimester and included a structured interview. Visit 3 was conducted following the hospitalization for labor and delivery and included a maternal interview, collection of maternal and infant biospecimens, as well as neurophysiological assessments of the newborn. Visit 4 occurred at a 6-month follow-up assessment after birth and included a comprehensive neurodevelopmental evaluation of the infant and postnatal environment. Participants were assigned into one of two study groups: PAE and controls (eligibility criteria are described below). Data included in this analysis was derived from the first two prenatal visits (Visits 1 and 2) resulting in a total sample size of 202 participants (65 in PAE and 137 in controls).

### Substance use measures

Alcohol use was evaluated utilizing repeated timeline follow-back (TLFB) interviews, targeted questions about episodic binge drinking, and a comprehensive panel of ethanol biomarkers. During TLFB interviews, patients were asked to report quantities of specific alcoholic beverages consumed each day during a 30-day period (Jacobson et al., 2002). Data utilized in this analysis included TLFB interviews which captured periconceptional period [2 weeks prior and 2 weeks after the last menstrual period (LMP)] and 30 days prior to enrollment (primarily second trimester). Ethanol biomarkers, collected at Visit 1, included gamma-glutamyl transferase (GGT), carbohydrate-deficient transferrin (%CDT), phosphatidylethanol (PEth), and urine ethyl glucuronide/ethyl sulfate (uEtG/EtS). Laboratory analysis of GGT was conducted at the TriCore Reference Laboratory (Albuquerque, NM, USA), analysis of %CDT was conducted at the Medical University of South Carolina (MUSC) laboratory (Charleston, SC, USA), and analyses of PEth and uEtG/uEtS were conducted at the US Drug Testing Laboratory (Des Plaines, IL, USA). Utilization of prospective repeated TLFB interviews and targeted questionnaires related to episodic binge drinking, coupled with a comprehensive battery of ethanol biomarkers, were shown to minimize self-report bias and more accurately estimate prevalence of exposure (Bakhireva and Savage, 2011; Bakhireva et al., 2021).

Subjects were classified into the PAE group if they reported more than minimal-risk alcohol use (>13 standard drink units [SDU]/month) or tested positive for at least one ethanol biomarker during pregnancy. Subjects reporting minimal alcohol use in the periconceptional period (no binge episodes around LMP and less than 13 drinks around LMP) and abstinence from alcohol use during pregnancy (per self-report, 0 binge episodes and 0 drinks and negative on all ethanol biomarkers at V1 and V2) were classified as controls. Participants were also asked to report the use of other substances, including tobacco use, at Visits 1 and 2 through a questionnaire based on the 2011 National Survey on Drug Use and Health (Substance Abuse and Mental Health Services Administration [SAMHSA], 2012). Self-reported substance use was validated via analysis of maternal urine samples collected during Visit 1. A Urine Drug Panel 7 (amphetamines, barbiturates, benzodiazepine, cannabinoid, cocaine, opiates and propoxyphene) was conducted at the United States Drug Testing Laboratories (Des Plaines, IL). Maternal substance use data reflects use during the time between LMP and Visit 2. To be classified as “no use” for a particular substance, participants had to be negative on both self-report and urine drug panel.

### Mental health and other measures

At Visit 1, participant’s socio-demographic characteristics (age, race, ethnicity, education, family income, marital status, primary language spoken at home), medical conditions (presence of chronic disease), and reproductive health characteristics (parity, prior pregnancy outcomes, planning of the current pregnancy, and any pregnancy complications) were ascertained.

Mental health outcomes of interest were evaluated during the Visit 2 interview through the following validated questionnaires: Perceived Stress Scale (PSS), Edinburgh Depression Scale (EDS), Generalized Anxiety Disorders-7 (GAD-7), and the Posttraumatic Stress Disorder Checklist for Diagnostic and Statistical Manual of Mental Disorders, Fifth Edition (PCL-5) (Cohen et al., 1983; Bergink et al., 2011; Simpson et al., 2014; Blevins et al., 2015). Additionally, the Visit 2 interview included assessment of social support by the Modified Medical Outcomes Social Support Survey (MOSS) and maternal childhood adversity by the ACE questionnaire (Sherbourne and Stewart, 1991; Osofsky et al., 2021). Data collected from MMH questionnaires primarily reflects mental health status at the time of assessment (third trimester) and up to 30 days prior depending on the screening tool (EDS, past 7 days; GAD-7, past 2 weeks; PSS and PCL-5, past month).

### Statistical analyses

Demographic and medical characteristics were summarized with means and standard deviations for quantitative variables and with frequencies (percentages) for categorical variables. Comparisons between controls and PAE participants were conducted using a *t*-test or non-parametric Mann-Whitney test for continuous variables and a Chi-square test or Fisher’s exact test for categorical variables, as appropriate. Normality of the distributions of quantitative variables was explored, to include quantile-quantile (Q-Q) plots of regression model residuals for MMH assessment measures.

Univariate linear regression models were constructed by regressing each MMH outcome variable on independent variables of interest under *biological domain* (PAE, cannabis use, medical conditions [hypertension/heart disease, diabetes, thyroid disorder, asthma or allergies, hepatitis, autoimmune disorder]), *social domain* (maternal education, marital status, social support in pregnancy, predominant language, and ACE), and *reproductive health domain* (pregnancy planning, current obstetric complications [bleeding during pregnancy, gestational hypertension/preeclampsia, gestational diabetes, placenta abnormalities], parity, and history of pregnancy loss). Initial multivariable models contained three main variables of interest (PAE, cannabis use, and social support) and other potential predictors delineated in the conceptual framework. Beyond the three main variables (included in all models), selection of the variables for the final model was determined using backward stepwise selection, with smaller Akaike information criterion (AIC) values indicating a better model fit (Vrieze, 2012). GAD-7 and PCL-5 measures were natural log-transformed to satisfy departures in model assumptions; all other MMH outcomes met model assumptions.

Additional *post hoc* analyses were conducted that investigated confounding of the relationship between MMH outcomes and cannabis use by other covariates. In these analyses, each MMH outcome was regressed first on cannabis use. Other covariates were then individually added to the regression to identify factors attenuating the significant relationship between MMH outcomes and cannabis use.

SAS statistical software (version 9.4; Cary, NC, United States) was used for statistical analyses. Analyses were two-tailed, and statistical significance was determined with an alpha level of 0.05; however, *p*-values of < 0.10 are also reported.

## Results

The sample included a sizable proportion of Hispanic/Latinx (63.4%) participants, 5.9% American Indian, and 12.4% patients who self-identified as “other” or were multi-racial. Participants were recruited across the entire span of socio-economic status: 40.6% had a college degree or higher, while 34.2% had a high school education or less; 35.6% reported family gross annual income as less than $30,000 and 28.2% more or equal to $70,000. The mean maternal age at enrollment was 29.5 ± 5.7. On average participants were recruited during the second trimester (25.1 ± 6.3 gestational weeks). No significant differences in socio-demographic characteristics were identified between PAE and controls (Table 1). Additionally, no significant differences between the groups were observed for reported medical conditions, pregnancy planning, and obstetric complications. Significant group differences were found for certain prior pregnancy outcomes, with a higher percentage of the PAE group being nulliparous (*p* < 0.01) and reporting prior pregnancy termination(s) (*p* < 0.01). The level of social support was higher among controls compared to PAE (84.0 ± 20.6 vs. 79.9 ± 21.5); however, differences did not reach statistical significance (*p* = 0.08). No differences in maternal ACE score were observed between PAE and controls (*p* = 0.32).

**TABLE 1:** Socio-demographic characteristics stratified by alcohol exposure (*N* = 202).

Characteristic	Control (*N* = 137)	PAE (*N* = 65)	*P*-value
	***N* (%)**	***N* (%)**	
**Race/Ethnicity:**			
White	90 (65.7)	51 (78.5)	0.20^1^
Black or African American	4 (2.9)	2 (3.1)	
American Indian or Alaskan Native	9 (6.6)	3 (4.6)	
Asian, Asian American or PI	8 (5.8)	0 (0.0)	
More than one race/Other	18 (13.1)	7 (10.8)	
Prefer not to report	8 (5.8)	2 (3.1)	
Hispanic/Latinx	85 (62.0)	43 (66.2)	0.57^3^
**Maternal education level:**			
High school or less	48 (35.0)	21 (32.3)	0.85^3^
Some college or vocational school	33 (24.1)	18 (27.7)	
College degree or higher	56 (40.9)	26 (40.0)	
**Partner status:**			
Married/Cohabiting	113 (82.5)	46 (70.8)	0.06^3^
**Maternal insurance:**			
No insurance	27 (19.7)	10 (15.4)	0.58^3^
Employer-based insurance	57 (41.6)	24 (36.9)	
Self-purchased insurance/Other	6 (4.4)	5 (7.7)	
Medicaid	47 (34.3)	26 (40.0)	
**Family gross annual income:**			
<$30,000	50 (36.8)	22 (34.4)	0.69^3^
$30,000–49,000	26 (19.1)	17 (26.6)	
$50,000–69,000	20 (14.7)	8 (12.5)	
≥$70,000	40 (29.4)	17 (26.6)	
**Primary language:**			
Spanish*	24 (17.5)	12 (18.5)	0.87^3^
**Pregnancy planning:**			
Planned/Unplanned	49 (35.8)	25 (38.5)	0.71^3^
**Chronic medical conditions:**			
Self-reported medical conditions:**	43 (31.4)	20 (30.8)	0.93^3^
Hypertension/heart disease	4 (2.9)	2 (3.1)	1.00^1^
Diabetes	6 (4.4)	2 (3.1)	1.00^1^
Thyroid disorder	6 (4.4)	2 (3.1)	1.00^1^
Asthma or allergies	17 (12.4)	7 (10.8)	0.74^3^
Hepatitis	0 (0.0)	1 (1.5)	0.32^1^
Autoimmune disorder	3 (2.2)	1 (1.5)	1.00^1^
Current obstetric complications:**			
Bleeding	10 (7.3)	4 (6.2)	1.00^1^
Gestational hypertension	7 (5.1)	4 (6.2)	0.75^1^
Diabetes	7 (5.1)	3 (4.6)	1.00^1^
Placenta previa	1 (0.7)	1 (1.5)	0.54^1^
Other***	12 (8.9)	4 (6.2)	0.51^3^
**Reproductive health**			
Parity: nulliparous	36 (26.3)	31 (47.7)	<0.01^3^
Previous pregnancy termination(s)	7 (5.2)	11 (17.5)	<0.01^3^
Prior pregnancy loss	25 (18.2)	17 (26.2)	0.20^3^
	**Mean ± SD**	**Mean ± SD**	
Maternal age at enrollment (years)	29.4 ± 5.6	29.7 ± 5.8	0.59^2^
Gestational age at enrollment (weeks)	24.8 ± 6.4	25.6 ± 6.3	0.34^2^
Social support: mMOSS	84.0 ± 20.6	79.9 ± 21.5	0.08^2^
Childhood trauma: ACE	2.3 ± 2.4	2.8 ± 2.9	0.32^2^

PI, Pacific islander; ACE, adverse childhood experiences; PAE, prenatal alcohol exposure; mMOSS, modified medical outcomes study social support survey.

*Patients were administered informed consent and all assessments in Spanish.

**Categories are not mutually exclusive.

***Other included: anemia, hematoma, nausea, arrhythmia, fibroids, fatigue, renal stones, gall bladder operation, palpitations, elevated A1c, and chronic back pain.

^1^Based on Fisher’s exact test.

^2^Based on Mann-Whitney test.

^3^Based on Chi-Square test.

With respect to maternal substance use, cannabis use was common with 18.8% prevalence across the entire sample. The prevalence of cannabis use was significantly higher in the PAE group compared to controls (35.4 vs. 10.9%, respectively; *p* < 0.0001; Table 2). Tobacco use was relatively low in both PAE (4.6%) and controls (1.5%) (*p* = 0.18). Among the controls, there were 19 participants who had some alcohol use after enrollment and were determined ineligible based on laboratory results (these participants were excluded in regression sensitivity analyses). In the PAE group, the average amount of alcohol per day in ounces (AA) across the periconceptional and prenatal periods (LMP to V2) was 0.2 AA, which is equivalent to approximately 3 standard alcohol drinks per week, an amount that is considered low-to-moderate level of exposure (Flak et al., 2014).

**TABLE 2:** Substance use patterns stratified by alcohol exposure (*N* = 202).

Substance use	Controls (*N* = 137)	PAE (*N* = 65)	*P*-value
Cannabis use, *n* (%)	15 (10.9)	23 (35.4)	<0.0001^3^
Tobacco use, *n* (%)	2 (1.5)	3 (4.6)	0.33^1^
**Periconceptional period**			
Any binge drinking episodes, *N* (%)	2 (1.5)	50 (76.9)	<0.0001^3^
# Binge drinking episodes, Mean ± SD	0.02 ± 0.19	3.88 ± 5.89	<0.0001^2^
AA/day, Mean ± SD	0.01 ± 0.02	0.58 ± 0.98	<0.001^2^
AA/drinking day, Mean ± SD	0.13 ± 0.37	2.01 ± 1.26	<0.001^2^
**Second trimester**			
Positive biomarkers > = 1, *n* (%)	19 (14)	15 (23.1)	0.12^3^
AA/day, Mean ± SD	0.00 ± 0.00	0.00 ± 0.01	0.07^2^
AA/drinking day, Mean ± SD	0.00 ± 0.02	0.03 ± 0.13	0.07^2^
**Third trimester**			
AA/day, Mean ± SD	0.00 ± 0.00	0.00 ± 0.01	0.01^2^
AA/drinking day, Mean ± SD	0.01 ± 0.08	0.06 ± 0.18	0.01^2^
Cumulative measures across periconceptional period and pregnancy*			
≥2 binge episodes, *n* (%)	1 (0.7)	43 (66.2)	<0.0001^3^
AA/day, Mean ± SD	0.00 ± 0.01	0.20 ± 0.33	<0.0001^2^
AA/drinking day, Mean ± SD	0.05 ± 0.14	0.70 ± 0.44	<0.0001^2^

AA, absolute alcohol (in ounces); 1 AA is equivalent to approximately 0.5 standard drinks; SD, standard deviation.

*Alcohol use from periconceptional period (1 month around LMP) to Visit 2 (third trimester).

^1^Based on Fisher’s exact test.

^2^Based on Mann-Whitney test.

^3^Based on Chi-Square test.

As shown in Table 3, participants in the PAE group had significantly higher scores for all MMH outcomes, i.e., PSS (*p* = 0.002), EDS (*p* = 0.003), GAD-7 (*p* = 0.007), and PCL-5 (*p* = 0.003) compared to controls.

**TABLE 3:** Maternal mental health outcomes in the third trimester stratified by alcohol exposure (*N* = 202).

Outcomes	Controls (*N* = 137)	PAE (*N* = 65)	*P*-value
	**Mean ± SD**	**Mean ± SD**	
Stress: PSS	12.4 ± 7.5	15.8 ± 7.2	0.002^1^
Depressive symptoms: EDS	5.6 ± 5.4	7.7 ± 5.5	0.003^1^
Anxiety: GAD-7	4.8 ± 4.9	6.5 ± 5.2	0.007^1^
PTSD symptoms: PCL-5	11.1 ± 14.0	16.6 ± 16.8	0.003^1^

PSS, perceived stress scale; EDS, Edinburgh depression scale; GAD-7, generalized anxiety disorder -7; PTSD, posttraumatic stress disorder; PCL-5, posttraumatic stress disorder checklist; PAE, prenatal alcohol exposure.

^1^Based on Mann-Whitney test.

Table 4 shows both univariate and multivariable associations between predictors of interest and MMH outcomes. It is important to note that while the three main predictors of interest (i.e., alcohol, cannabis, social support) were kept constant in all models, the conclusive list of covariates in final, parsimonious models varied based on specific MMH outcome, thus direct comparisons of regression coefficients should not be made.

**TABLE 4:** Predictors of maternal mental health during pregnancy: results of univariate and multivariable linear regression analyses.

Predictors	PSS	GAD-7	EDS	PCL-5
	**β (SE)**	**β (SE)**	**β (SE)**	**β (SE)**
**Biological factors:**				
**Alcohol: PAE vs. controls**				
Univariate	3.52 (1.13)**	0.37 (0.13)**	2.00 (0.82)*	0.59 (0.19)**
Multivariable	2.40 (1.05)*	0.23 (0.12)	1.11 (0.74)	0.35 (0.17)*
**Cannabis: any vs. none**				
Univariate	4.44 (1.34)**	0.44 (0.16)**	3.18 (0.97)**	0.79 (0.22)***
Multivariable	0.03 (1.40)	−0.05 (0.16)	−0.19 (0.98)	0.08 (0.22)
**Medical conditions: any vs. none**				
Univariate	−2.97 (2.26)	−0.23 (0.27)	−2.50 (1.63)	0.05 (0.38)
Multivariable	−4.22 (2.00)*	−0.40 (0.22)	−3.53 (1.40)*	–
**Social factors:**				
**Social support score (1-unit increase)**				
Univariate	−0.15 (0.02)***	−0.02 (0.00)***	−0.12 (0.02)***	−0.03 (0.00)***
Multivariable	−0.12 (0.02)***	−0.01 (0.00)***	−0.10 (0.02)***	−0.02 (0.00)***
**Maternal ACE (1-unit increase)**				
Univariate	0.95 (0.20)***	0.11 (0.02)***	0.77 (0.14)***	0.19 (0.03)***
Multivariable	0.61 (0.22)**	0.09 (0.03)***	0.55 (0.15)***	0.14 (0.03)***
**Primary language: Spanish vs. English**				
Univariate	−3.16 (1.40)*	−0.40 (0.17)*	−1.81 (1.02)	−0.34 (0.23)
Multivariable	−3.59 (1.26)**	−0.34 (0.15)*	−2.18 (0.89)*	–
**Maternal education: College vs. ≤ high school**				
Univariate	0.19 (1.24)	0.16 (0.15)	−0.05 (0.90)	0.12 (0.20)
Multivariable	–	0.33 (0.14)*	–	0.44 (0.18)*
**Some college/vocational vs. ≤ high school**				
Univariate	2.12 (1.42)	0.25 (0.17)	1.11 (1.03)	0.67 (0.23)**
Multivariable	–	0.19 (0.14)	–	0.59 (0.20)**
**Marital status: with partner vs. single**				
Univariate	−2.11 (1.32)	−0.29 (0.16)	−1.29 (0.95)	−0.53 (0.22)*
Multivariable	–	–	–	–
**Obstetric complications: any vs. none**				
Univariate	−0.21 (1.46)	−0.04 (0.17)	−0.49 (1.06)	0.21 (0.24)
Multivariable	–	–	–	–
**Reproductive health factors:**				
**Parity**				
Univariate	0.26 (0.43)	−0.04 (0.05)	0.15 (0.31)	−0.06 (0.07)
Multivariable	–	–	–	–
**History of a pregnancy loss: any vs. none**				
Univariate	2.71 (1.31)*	0.37 (0.15)*	1.93 (0.95)*	0.34 (0.22)
Multivariable	–	–	–	–
Multivariable model F statistic	12.30	9.41	15.00	15.98

*p < 0.05;

**p < 0.01;

***p < 0.001.

Results of the final parsimonious model is presented for each outcome. Covariates without estimates listed in multivariable row were not included in the final model.

With respect to *biological factors*, PAE (*p* < 0.05) and cannabis use (*p* < 0.05) were associated with higher (worse) scores for all MMH outcomes in univariate analyses. In the final multivariable models, PAE remained as a significant independent predictor of lower PSS (β = 2.4 ± 1.05), GAD-7 (β = 0.23 ± 0.12), and PCL-5 (β = 0.35 ± 0.17; all *p’s* < 0.05). Associations between cannabis use and MMH variables, however, became non-significant after adjustment for other factors. Medical conditions were not associated with MMH outcomes in univariate models but became significant in multivariable models for PSS and EDS.

With respect to *social factors*, social support was inversely associated with all MMH outcomes (all *p’s* < 0.001) in both univariate and multivariable analyses, meaning that higher level of social support was associated with better MMH outcomes. Higher maternal ACE score, indicating wider scope of childhood adversity, were associated with worse scores for all MMH outcomes in both univariate (all *p’s* < 0.001) and multivariable (all *p’s* < 0.01) analyses. Spanish as the primary household language was associated with lower PSS and GAD-7 scores in univariate analyses and lower PSS, GAD-7, and EDS in multivariable analyses (all *p’s* < 0.05). Having a partner during pregnancy was associated with lower PCL-5 scores in univariate analysis, but not in the final multivariable model. Some college or vocational school was associated with higher PCL-5 scores (*p* < 0.01) in both univariate and multivariable analyses, and having a college degree was associated with higher PCL-5 scores and with higher GAD-7 scores in multivariable analyses (*p* < 0.05).

With respect to *reproductive health factors*, history of pregnancy loss was associated with higher PSS, GAD-7, and EDS scores (*p* < 0.05) in univariate analyses, but became non-significant after adjustment for other factors. Parity was not significantly associated with MMH outcomes in univariate or multivariable models, nor was the experience of one or more obstetric complications (bleeding, gestational hypertension, diabetes, or placenta previa) during the current pregnancy.

**FIGURE 2:**
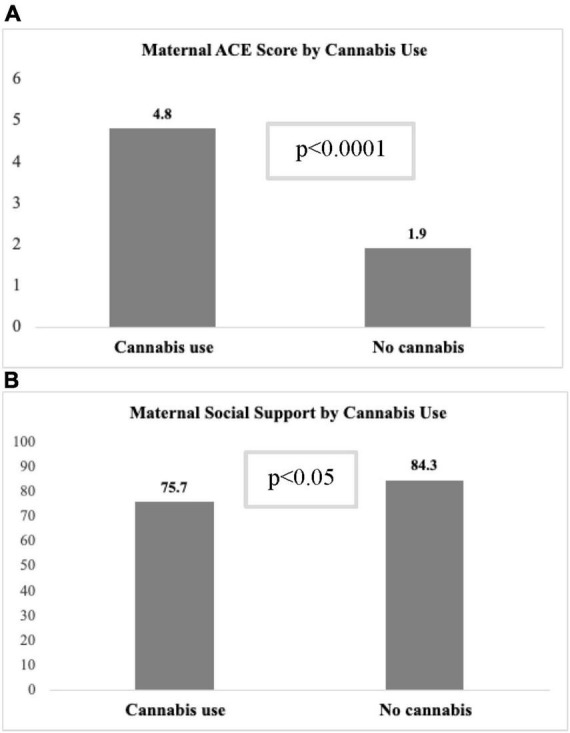
**(A)** Mean maternal ACE and **(B)** social support scores by prenatal cannabis exposure.

Because cannabis had a significant association with all MMH outcomes in univariate models but not in multivariable models, additional analyses were conducted to identify covariates that impacted association between cannabis use and MMH outcome scores (data not shown). The variable with the strongest attenuation effect was the ACE score. The mean ACE score was significantly higher among participants who used cannabis in pregnancy compared to those with no use (4.8 ± 2.9 vs. 1.9 ± 2.2, *p* < 0.001; Figure 2A). In multivariable regression models that only included cannabis use and ACE score, there was a significant relationship between ACE score and all MMH outcomes (*p* < 0.001), but not between cannabis use and MMH outcomes (all models, *p* > 0.20) indicating a strong confounding effect of ACE score. The interaction between ACE score and cannabis use, however, was not significant in any models that included adjustment for other covariates. Other variables that attenuated the relationship between cannabis use and MMH outcomes were PAE and level of social support. Among participants who used cannabis during pregnancy, a significantly higher percentage were classified into the PAE group vs. controls (60.5 vs. 39.5%, *p* < 0.0001). Additionally, social support score was significantly lower (75.7 ± 24.3 vs. 84.3 ± 19.8, *p* = 0.02) among cannabis users compared to those who abstained from cannabis (Figure 2B).

## Discussion

Results of this study demonstrate that the mean scores for maternal stress, and symptomatology of depression, anxiety and PTSD were substantially higher in pregnant persons who used alcohol during pregnancy compared to controls. To our knowledge, this is the first study which comprehensively examines the effect of the two most commonly used substances in pregnancy (alcohol and cannabis), maternal childhood trauma, pregnancy-related factors, and potentially modifiable resilience factors (social support, enculturation) with respect to multiple mental health outcomes in the same cohort. The innovation is also driven by our focus on low-to-moderate alcohol use in pregnancy with detailed characterization across periconceptional period and later in pregnancy using prospective repeated self-report measures and a comprehensive battery of ethanol biomarkers. Interestingly, the level of social support was lower, and the prevalence of concurrent cannabis use was considerably higher among participants who used alcohol compared to controls. Alcohol use and maternal ACEs were associated with worse scores for all mental health outcomes, while higher level of social support and Spanish as the primary household language had a strong protective effect (associated with lower scores) on mental health outcomes. These findings highlight the complex interplay among polysubstance use, social support, and MMH in pregnancy.

The independent association between PAE and impaired mental and emotional wellness during pregnancy is in accord with previous observations. Prior research demonstrated that alcohol use in pregnancy had been consistently associated with higher odds of experiencing impaired perinatal mental health (Zhao et al., 2017). It is important to note that most prior studies focused on perinatal depression (Pentecost et al., 2021). While anxiety and PTSD have independently demonstrated high comorbidity with depression, only a few studies examined the individual effect of PAE on these MMH outcomes. Findings from those studies are congruent with our report in demonstrating an independent association between PAE and perinatal symptoms of anxiety and PTSD (Leis et al., 2012; Prevatt et al., 2017).

Prior literature provides somewhat heterogeneous results with respect to an association between PAE and maternal depression. A prospective cohort study found that maternal alcohol use was associated with depressive symptoms and increased life stress during pregnancy (Zuckerman et al., 1989). Similarly, in a randomized clinical trial that investigated the efficacy of a brief intervention to decrease PAE among pregnant women recruited early in their prenatal care course, it was found that substance use predicted maternal depression co-occurring with prenatal alcohol use (Rubio et al., 2008). However, other studies found no association between PAE and depressive symptoms (Pajulo et al., 2001; Rubio et al., 2008; Leis et al., 2012). Interestingly, the most robust association with respect to PAE in the present study was observed for stress (PSS) and anxiety-related outcomes (GAD-7 and PCL-5) rather than depressive symptoms. In a retrospective cohort study, it was found that women who used alcohol and tobacco during pregnancy had significantly greater odds of reporting prior history of mental illness including anxiety, PTSD, depression, and/or depressive symptoms during pregnancy (Zhao et al., 2017). Thus, the direction of association in terms of prior mental health issues leading to substance use in pregnancy or substance use worsening mental health outcomes remains debatable.

Our study found a significant effect of prenatal cannabis use on MMH outcomes in univariate models and after controlling for the effect of concurrent alcohol use. However, results became non-significant after adjusting for maternal ACE score, predominant language, and social support during pregnancy, suggesting that those factors have a stronger underlying effect on mental health. In fact, additional analyses investigating relationships between MMH outcomes and cannabis use demonstrated that ACE score was substantially higher among persons who used cannabis during pregnancy compared to non-users. Several prior studies found that higher maternal ACE score was associated with increased risk for PAE and prenatal cannabis use (Chung et al., 2010; Frankenberger et al., 2015; Smith et al., 2016) as well as perinatal symptoms of depression and PTSD (Osofsky et al., 2021). Moreover, prenatal cannabis use was associated with maternal depression, anxiety and higher rates of generalized trauma (Young-Wolff et al., 2019). One study found that while the total ACE score was unrelated to maternal substance use, ACE subtypes of childhood mistreatment and household familial dysfunction were positively associated with increased risk for maternal cannabis use and PAE, respectively (Osofsky et al., 2021). Some variability in findings regarding ACE score and maternal substance use might be attributed to disparities in patient characteristics and confounders that were accounted for in the analyses. Prior studies have reported maternal stress as an important attenuating factor in the association between ACE score and prenatal substance use (Osofsky et al., 2021).

Social support during pregnancy is increasingly being recognized in the literature as an important protective and potentially modifiable factor affecting MMH outcomes. In our analysis, a higher level of social support was associated with fewer symptoms of maternal anxiety, depression, stress, and PTSD. Consistent with our report, a systematic review and meta-analysis that included 67 studies with a combined total of 64,449 pregnant persons demonstrated that low social support during pregnancy was associated with an increased risk of maternal anxiety and depression (Bedaso et al., 2021). Among the 15 studies included in this meta-analysis which assessed the association between social support and antenatal depression, 14 identified a significant inverse relationship and no significant correlation was identified in 1 (Bedaso et al., 2021). Among 8 studies which assessed the interplay between social support and pregnancy-related anxiety, a significant association emerged in 7 of these studies and 1 could not be assessed due to insufficient evidence (Bedaso et al., 2021). Prior analyses have additionally demonstrated an association between low social support index and maternal substance use disorders, including alcohol use disorder (Pajulo et al., 2001).

Community-based outreach programs have been proposed as a means to augment maternal social support and improve MMH outcomes, although, success has been variable. A randomized control trial evaluating repeated nurse home visits demonstrated reduced levels of depression and stress in the intervention group (Goldfeld et al., 2021). A similar study evaluating repeated home visits conducted by a social worker found that the intervention may result in elevated maternal stress levels (Lever et al., 2019). A systematic review examining multi-modal group parenting programs found no significant improvement in maternal stress, anxiety or depression (Waldrop et al., 2021). At UNM, the organization home to the present study, several institution-based initiatives have been implemented, including a volunteer-doula birth companion program as well as a specialized, multidisciplinary perinatal psychiatric clinic (Del Fabbro, 2016; Kivlighan et al., 2022). The ACOG recommends pharmacotherapy as the first-line method of treatment for depression, anxiety and PTSD in pregnant and postpartum patients. Adjunctive therapy may include behavioral health services, promotion of self-care practices (e.g., balanced nutrition, mindfulness, exercise, substance avoidance), and assessment of structural and social determinants of health (ACOG Committee on Clinical Practice Guidelines, 2023).

Interestingly, we observed a strong protective effect of Spanish as the primary language spoken at home on MMH. The potential explanation for this phenomenon is two-fold. First, language preference is a proxy of a birthplace and multiple sociocultural factors which affect maternal health and wellbeing in general. In fact, a so-called Hispanic paradox refers to a well-documented phenomenon in which Hispanic/Latinx immigrants exhibit better overall health, including mental health despite socio-economic risk factors (Alarcón et al., 2016). Longer time residing in the US and associated acculturation have been associated with increased rates of mental health disorders and increased prevalence of substance use among Hispanic immigrants (Alarcón et al., 2016). We have previously reported that pregnant Latinx patients who are born in the US and those who primarily speak English at home engage in much riskier alcohol consumption behaviors compared to foreign-born Latinx patients and Spanish-speaking patients (Bakhireva et al., 2009). Thus, socio-cultural factors in the context of close-knit community, rather than country of nativity *per se*, might be a crucial protective factor. It is important to acknowledge that the Spanish-speaking group in our study does not represent a homogeneous group; however, most Spanish-speaking patients in UNM satellite clinics are of Mexican descent.

This study has several potential limitations to be considered. First, our results were based on screening tools for stress, depression, anxiety, and PTSD rather than clinical diagnosis of these conditions. It is important to acknowledge, however, that validated, clinically relevant scales were used to assess emotional and mental wellness in pregnancy. Second, while multiple risk and protective factors were evaluated in the current study and the analytical approach was guided by a well-developed conceptual framework and comprehensive literature review, we acknowledge additional factors (e.g., nutrition, physical activity, systemic racism) known to affect mental health which were not evaluated in this study. Third, while validated scales were used to assess MMH, and TLFB interviews are the current “gold standard” to assess substance use, the role of social desirability biases cannot be excluded. However, it should be noted that self-reported alcohol and substance use was validated by a panel of biomarkers. The combined use of repeated, prospective TLFB interviews, targeted questions about binge drinking episodes, and a broad panel of ethanol biomarkers is the most comprehensive approach to characterize PAE (Bakhireva and Savage, 2011; Bakhireva et al., 2021). Further, we have previously reported high concordance between disclosure of less stigmatized substances, such as cannabis, and urine drug tests (Garg et al., 2016; Page et al., 2022). Fourth, maternal involvement in substance use and/or mental health treatment was not included in the analytical framework. As described in the methods, co-exposure with opioids, cocaine, and methamphetamines were exclusionary criteria, and no participants were receiving treatment for alcohol/substance use. This paper focused on risk and resiliency factors that affect MMH outcomes rather than on the treatment of disordered MMH. Future studies should examine a combined effect of non-pharmacologic (e.g., social support, trauma-focused behavioral interventions) and pharmacologic interventions on MMH outcomes. Lastly, although our findings identified a strong association between maternal substance use and impaired MMH, the present study does not address the temporality of this relationship.

Unique strengths of the study include prospective nature of the study and a comprehensive evaluation of prenatal exposures (by prospective repeated interviews and a comprehensive panel of biomarkers) and mental health outcomes. We are not aware of prior studies which assessed two most common prenatal exposures (alcohol and cannabis), socio-cultural factors, and maternal history ACE with respect to maternal emotional and mental wellness in the same study population. Additionally, a unique feature of this study is our focus on low-to-moderate levels of alcohol use. While prior studies focused on a high level of exposure, or did not report the level of PAE (Chung et al., 2010; Frankenberger et al., 2015; Smith et al., 2016), our study filled a gap in knowledge by demonstrating a robust effect of even lower level PAE on MMH outcomes. Lastly, the conceptual framework, adapted from the biopsychosocial model, provides a practical guide to interpreting the determinants of MMH. Future studies may strengthen the present framework by incorporating additional factors which have distinct implications for mental health in the setting of pregnancy (e.g., birth trauma and postnatal PTSD, intimate partner violence, prenatal psychiatric medication use, sleep).

## Conclusion

In conclusion, this study identified key risk and protective factors related to MMH outcomes among the constellation of contributing factors. Of note, it is particularly concerning that even low-to-moderate alcohol consumption in the perinatal period imposes a significant negative impact on MMH. Additionally, this analysis demonstrates a significant protective effect of socio-cultural factors on maternal mental and emotional wellbeing. These findings underscore the need for diligent evaluation of maternal substance use patterns both during and before pregnancy. Additionally, while clinical guidelines recommend MMH screening, significant gaps persist emphasizing the need for repeated screening throughout the prenatal care course. The impact of social support on MMH validates its relevance in discussions between maternal health professionals and patients. Furthermore, social support may act as a leverage point for targeted community-based and institution-based programs aiming to improve MMH outcomes. Policymakers and other pertinent stakeholders should invest in the development of evidence-based interventions that promote resiliency factors while mitigating risk factors in order to ultimately improve both maternal and child health outcomes.

## Data availability statement

The datasets generated and/or analyzed during the current study are not publicly available due to lack of data sharing acknowledgement of de-identified data in the consent and IRB protocol. The request for data sharing can be considered on a case-by-case basis with a formal data sharing agreement between institutions.

## Ethics statement

The studies involving humans were approved by the University of New Mexico Human Research Review Committee. The studies were conducted in accordance with the local legislation and institutional requirements. The participants provided their written informed consent to participate in this study.

## Author contributions

FW: Conceptualization, Investigation, Methodology, Visualization, Writing – review and editing. HN: Conceptualization, Methodology, Writing – original draft, Writing – review and editing. JD: Data curation, Formal Analysis, Investigation, Writing – review and editing. XM: Data curation, Formal Analysis, Investigation, Writing – review and editing, Methodology, Software. MR: Data curation, Investigation, Supervision, Writing – review and editing, Methodology, Validation. LB: Conceptualization, Funding acquisition, Supervision, Writing – review and editing, Investigation, Methodology, Project administration, Resources.
